# Recurrent Pregnancy Loss: Immunological aetiologies and associations with mental health

**DOI:** 10.1016/j.bbih.2024.100868

**Published:** 2024-09-19

**Authors:** Riddhi A Laijawala

**Affiliations:** Department of Psychological Medicine, Institute of Psychiatry, Psychology & Neuroscience, King's College London, United Kingdom

## Abstract

Recurrent pregnancy loss (RPL) is an obstetric condition estimated to affect 2–4% of childbearing individuals globally. Due to its varied nature, medical societies globally differ in their diagnostic criteria. Its aetiologies are numerous, ranging from anatomic abnormalities to endocrine and immunological factors. Autoimmune factors can attribute to approximately 20% of cases and include dysregulation of immune cells, cytokine production and antiphospholipid syndrome. Treatment pathways vary by aetiology; however, many cases remain unexplained, adding an additional level of complexity to this condition. Due to its recurrent nature, this type of pregnancy loss has profound impacts on mental health during subsequent pregnancies. While some aspects of RPL have been widely investigated, there continues to be a gap in research, such as its impacts on non-birthing parents and specific sociodemographic groups.

## 1 Introduction

Recurrent pregnancy loss (RPL), also known as recurrent miscarriage or habitual abortion (Ford and Schust, 2009) is an obstetric complication referring to the loss of more than one pregnancy. This includes both, embryonic (occurring prior to 10 weeks' gestation) and foetal losses (occurring after 10 weeks’ gestation) (Dimitriadis et al., 2020; El Hachem et al., 2017), and excludes ectopic and molar pregnancies, as well as implantation failures (Sultana et al., 2020).

Notably, no one definition of RPL exists, as medical societies differ in their criteria of the number of losses, gestational age, and pregnancy type. In the UK, the Royal College of Obstetricians and Gynaecologists uses the term recurrent miscarriage as three or more miscarriages in the first trimester (Regan et al., 2023; Dimitriadis et al., 2020). As per this definition, recurrent miscarriage is estimated to affect 1% of women (Regan et al, 2023). In contrast, the American Society for Reproductive Medicine defines RPL as two or more clinical pregnancy losses (Dimitriadis et al., 2020). Literature published by the society estimates that fewer than 5% of women experience two consecutive losses, while only 1% would experience three or more (American Society for Reproductive Medicine, 2012). However, the European Society of Human Reproduction and Embryology (ESHRE) in their guidelines recommends the use of ‘recurrent pregnancy loss’ to define repeated pregnancy losses (The ESHRE Guideline Group on RPL, 2018), and the term ‘recurrent miscarriage’ for the repeated loss of confirmed intrauterine pregnancies (Dimitriadis et al., 2020). Common risk factors include, but are not limited to advanced maternal age, chromosomal abnormalities, uterine anomalies, endocrine disturbances, immunological diseases, and thrombophilia (Sultana et al., 2020).

Additionally, the lack of consensus in deciding the number of losses to confirm the diagnosis of RPL has led clinicians to debate the point at which an investigation should be conducted to identify causes and choice of treatment. van Dijk and colleagues in 2020 completed a meta-analytic review of studies, and based on results, suggested that workups should begin after two losses (van Dijk et al., 2020).

This review will explore and synthesize evidence about (a) the role of the immune system in recurrent pregnancy loss and its treatment, (b) the impact of recurrent pregnancy loss on mental health and (c) understand the current literature on maternal foetal attachment after recurrent pregnancy loss.

## 2 Aetiologies of RPL

Recurrent pregnancy loss is a complex reproductive complication, and aetiologies are varied. 2–4% of recurrent pregnancy losses are associated with genetic aetiologies such as single cell defects. Anatomic abnormalities such as intrauterine adhesions and polyps account for 10–15% of RPL cases. Endocrine aetiologies (associated with 17–20% of cases) include polycystic ovarian syndrome and diabetes mellitus, while certain infections such as mycoplasma and herpes simplex virus are suspected to play a role in 0.5%–5% RPL occurrence (Ford and Schust, 2009). In addition to these, the role of the immune system has been investigated as a contributing factor to RPL. It is estimated that 20% of RPL cases can be attributed to autoimmune aetiologies (Ford and Schust, 2009). This means a large number of recurrent pregnancy losses remain unexplained, and this adds another layer of complexity, as treatment types differ according to the reason for loss. When RPL remains unexplained, or treatment pathways are unclear, management of the condition is challenging. This is because additional and excessive testing is prescribed by healthcare professionals, which can worsen anxiety in the couple when no aetiology or treatment is identified.

### 2.1 Role of the immune system in RPL

In normal/healthy pregnancies, the immune system is tightly regulated to prevent rejection of the foetal allograft, while protecting the gestational parent and unborn baby from pathogens (Sawyer, 2021). For example, certain pro-inflammatory cytokines are elevated in the blood throughout pregnancy. In contrast, anti-inflammatory cytokines potentially have reduced levels in the first half of pregnancy (Sawyer, 2021) Therefore, as the immune system is a key mechanism to support pregnant females and their developing foetuses (Morelli et al., 2015), its dysregulation and associated mechanisms can potentially be risk factors in the occurrence of RPL.

One of these mechanisms is the contrasting level of immune cells seen in females with and without history of RPL. Levels of Th1 cells are slightly increased in females with RPL, as compared to normal pregnancies, wherein-they are slightly decreased (Ali et al., 2020). On the other hand, Th2 cells are slightly decreased in females with RPL history (Ali et al., 2020). Regulatory T (Treg) cells, which have been postulated to play a role in RPL have reduced frequency and function as compared to healthy females (Ghaebi et al., 2017; Ali et al., 2020). Th17 cells are in contrast significantly increased in those affected, as compared to females with normal pregnancy (Ali et al., 2020).

The dysregulation of Natural Killer (NK) cells has also been postulated to play a role in RPL (Dosiou and Giudice, 2005). Indeed, NK cells emerge as among the most pivotal cells in maternal-foetal immune tolerance, through the interaction of maternal killer cell immunoglobulin type receptors, and foetal leukocyte antigens (Li et al., 2023). Laird et al. have discussed increased numbers of CD56^+^ NK cells in the peripheral blood of women with RPL during pregnancy, compared with healthy controls, in whom CD56^+^ NK cell activity decreases in the first trimester (Laird et al., 2003). In the placental tissue of women with RPL however, there are fewer decidual CD56^+^ NK cells (Laird et al., 2003). In contrast, Katano and colleagues sought to investigate the role of preconception peripheral blood natural killer cell (pNK) activity in women with RPL. They found that high pNK cell activity was not an independent risk factor in subsequent pregnancy losses (Katano et al., 2013).

Certain immune-associated genes have been identified to be associated with RPL. Specifically, the rs910352T allele, and the rs2070777AA genotype of the SERPINA4 gene are relevant to the risk of experiencing RPL (Li et al., 2023). The SERPINA4 gene, which encodes kallistatin, is involved in regulating the expression of numerous genes, and literature has identified that it is the polymorphisms of SERPINA4 which are associated with pregnancy loss (Che et al., 2021).

Evidence has also shown that variations in cytokine production exist. Daher et al. (2004) sought to investigate cytokine production in women with RPL (N = 29) compared with healthy controls without history of miscarriage (N = 27). They found higher levels of IFNγ and increased TNF⍺ production in the RPL group compared with the control group. Similarly, Giannubilo et al. (2012) investigated inflammatory cytokine expression patterns in women with RPL, when compared with healthy controls. Using trophoblastic tissue obtained from uterine evacuation, they found (1) downregulation of Transforming growth factor beta 3 (TGF-β3) and Interleukin 25 (IL-25), and (2) upregulation of Interleukin 2 receptor alpha (IL-2RA). Ali et al. (2020) reviewed the current evidence base, and concluded that (a) TNFɑ, TNFβ, IFNγ, IL-1 and IL-2, are slightly increased in women with RPL, (b) IL-4, IL-6 which are slightly increased in people with normal pregnancies are in fact slightly decreased in RPL patients, (c) IL-17 and IL-2 are significantly increased, and finally, (d) TGFβ is significantly decreased in women with RPL (Ali et al., 2020). In a case-control study undertaken by Comba and colleagues, blood and endometrial tissue samples were collected from 21 women with RPL and 20 healthy controls. They specifically found that blood and tissue levels of IL-12, IL-18, and IFNγ were significantly higher, while levels of leukaemia inhibitory factor (LIF) and migration inhibitory factor (MIF) were significantly lower in the RPL group (Comba et al., 2015).

Certain immune checkpoints have been investigated in the context of RPL. Marozio et al. (2023) studied the gene expression and quantitative tissue protein levels of the following immune checkpoints: CD276, Cytotoxic T-lymphocyte -associated antigen−4 (CTL-4), and lymphocyte activation gene-3 (LAG-3) in endometrial samples from 27 women with unexplained RPL, compared with controls. They found that CD276 gene expression and protein tissue levels were significantly lower in the endometrium of the RPL group, while CTL-4 and LAG-3 were significantly higher (Marozio et al., 2023).

As the presence of positive antinuclear antibodies (ANA) is considered a typical feature of autoimmunity (Chen et al., 2020), this biomarker has been investigated in the context of RPL. A meta-analysis of 21 studies and 5038 participants found that ANA positivity was associated with a risk of RPL, with a positive rate of 22% in the RPL group, compared with 8.3% in the healthy control group (Chen et al., 2020). These results are in line with another meta-analysis by Cavalcante and colleagues. Drawing results from seven case-control studies, the authors found that the frequency of positive ANA was higher in the group with a history of RPL (20.6%), when compared with healthy controls (6.7%) (Cavalcante et al., 2020).

Literature has additionally highlighted the role of the human leukocyte antigen (HLA) molecule in the occurrence of recurrent pregnancy loss (Bansal, 2010; Grimstad and Krieg 2016). Indeed, its unique expression pattern suggests that it is a key player in the survival of the foetus, and a healthy pregnancy (Abbas et al., 2004). In a sample of Caucasian women experiencing RPL, Thomsen et al. (2021) found that the HLA-DRB1∗07 allele was significantly increased, as compared to healthy controls. Abbas and colleagues identified another allele, HLA-G∗010103, which was higher in a sample of women from India experiencing RPL. They also found that the alleles present in women experiencing RPL, HLA-G∗010105 and HLA-G∗010108 were in contrast, totally absent in healthy controls (Abbas et al., 2004). This is particularly relevant to reproductive health, as the HLA-G is a vital component in the modulation of the maternal immune system during pregnancy, and is associated with the maternal acceptance of the semi allogenic foetus (Hviid, 2006)

Apart from an increased risk of RPL, studies have identified an association between increased HLA expression and obstetric outcomes. For example, in a cross-sectional study, Stout and colleagues found that higher levels of HLA-G expression are associated with preterm birth (Stout et al., 2015). Especially in the case of pregnancy following RPL, these obstetric outcomes can pose as significant risks to both, the gestational parent as well as the offspring.

Another immunological factor postulated to underlie RPL occurrence is Antiphospholipid antibody syndrome (APS), a disorder of the immune system known to increase the risk of blood clots (NHS UK, 2022) which has been associated with adverse obstetric outcomes including RPL. In fact, literature portrays APS as one of the most significant treatable factors in the context of RPL (Rai and Regan, 2006). A 2017 meta-analysis sought to understand the association between RPL and antiphospholipid antibodies (Santos et al., 2017). They found that lupus anticoagulant (LA), anti-β2-glycoprotein I antibodies (aβ2GPI0), antiphosphatidylserine (aPS), and anticardiolipin antibodies (aCL) showed a relationship with recurrent miscarriage. This was in line with a review conducted by Vinatier and colleagues, who discussed that between 7 -25% of RPL cases have APS as a primary risk factor and highlight that miscarriages might occur due to antiphospholipid antibody including trophoblast dysfunction (Vinatier et al., 2001). It is important to review obstetric outcomes following RPL in those with a diagnosis of APS. Specifically, increased risk of neonatal mortality, delivery of small for gestational age infants, and pre-eclampsia, are obstetric outcomes associated with APS (Walter et al., 2021). Research has also highlighted that aβ2GPI0 is the antibody associated with incidence of preeclampsia, intrauterine growth restriction, and stillbirth (Saccone et al., 2017). The risk of obstetric complications increases when more than one antiphospholipid antibody is pregnant (Saccone et al., 2017). These obstetric outcomes can significantly impact subsequent pregnancies after experiencing RPL.

The above-mentioned immune-related factors contributing to the occurrence of RPL are all associated with the female, and current research evaluating the role of male mechanisms is scarce. Muncey et al. (2024) reviewed potential paternal/male mechanisms underpinning pregnancy loss. Of note, they highlighted advanced paternal age to be associated with higher risk of RPL. In terms of biological mechanisms, they also discussed studies showing that men whose partners experienced RPL had higher DNA fragmentation index, with elevated levels of sperm fragmentation.

This evidence is in line with the ideology that RPL is indeed a “disease of inflammation and coagulation”, as highlighted by Kwak Kim and colleagues (2009). Primarily, it is the dysregulation of immune cells and the cytokines they produce, which play a role in RPL due to immunological factors.

It is vital to note that these above-mentioned factors, i.e. endocrine disturbances, uterine anomalies, genetic, and immunological aetiologies contribute to a small number of explained causes in RPL occurrence. Still, causes of RPL are unexplained in less than half of the cases (El Hachem et al., 2017). This complicates the formulation of treatment strategies and highlights the need to create personalized interventions for couples experiencing RPL, in order to protect subsequent pregnancies, with the ultimate goal to be a live birth of an infant.

#### 2.1.1 Treating recurrent pregnancy loss due to immunological aetiologies

Given the complexity of RPL as a reproductive disorder with varied aetiologies, established treatment types differ. Genetic counselling, donor gametes, and IVF with preimplantation genetic diagnoses are suggested for RPL due to genetic aetiologies (Ford and Schust, 2009).

For autoimmune disease like APS, low dose aspirin is suggested for women without history of systemic autoimmune disease (Ford and Schust, 2009), as it seems to be a potent stimulator of IL-3, known to be reduced in APS related pregnancy losses (Vinatier et al., 2001).

Low molecular weight Heparin is another treatment mechanism suggested in the context of RPL due to APS, and studies have shown that with therapeutic dosage, 93% of pregnancies in people with APS led to a live birth (Vinatier et al., 2001). Heparin is known to have anticoagulant effects, and primarily, it is reported to inhibit the binding of antiphospholipid antibodies to their target in vitro. This effect is subsequently protective for trophoblastic phospholipids, which thereby promote successful early pregnancy implantation (Vinatier et al., 2001).

In addition, Intravenous immunoglobulin (IvIg) has been used and proven effective to treat RPL due to immunological factors (Kwak Kim et al., 2009), as it inhibits the production of pro-inflammatory cytokines, and enhances the proportion of cells producing anti-inflammatory cytokines.

Finally, corticosteroid drugs like prednisone have been implemented as a treatment mechanism due to their ability to suppress cytotoxic activity of NK cells, however, they have been associated with pregnancy related side effects like gestational diabetes (Kwak Kim et al., 2009).

The variation in definition and non-consensus in diagnosis, coupled with multiple aetiologies makes RPL a complex reproductive condition to treat. In order to ensure that the most timely and appropriate treatment strategy is implemented, consensus will have to be reached by medical societies. Personalized investigations regarding risk factors can be highly beneficial to ensure healthy subsequent pregnancies.

## 3 Recurrent pregnancy loss and mental health

As well as being a complex biological process, recurrent pregnancy loss is an intense emotional experience which can have negative consequences on psychosocial outcomes. Research shows that in the first six months following a pregnancy loss, women are at an elevated risk of developing depressive symptomology (Klier et al., 2002). When loss is of a recurrent nature, depressive symptomology can intensify into the development of Major Depressive Disorder.

Experiencing RPL can lead to high levels of pregnancy specific stress and anxiety in subsequent pregnancies due to the fear of experiencing another loss. Furthermore, a varied range of research shows that prenatal anxiety is associated with higher risk of preterm birth, lower birth weight, and smaller head circumference (Grigoriadis et al., 2018). This evidence underpins the importance of addressing maternal mental health, especially among pregnant people with a history of RPL. A growing body of literature focuses on the mental health of females after experiencing pregnancy loss in general. For example, Lok and colleagues conducted a longitudinal study investigating psychological outcomes following pregnancy loss (Lok et al., 2010). They found that 55% of women with pregnancy loss scored high on the General Health Questionnaire (GHQ) (a measure to assess a diagnosable psychiatric disorder) (Goldberg and Hillier, 1979) and 26.8% scored high on the Beck Depression Inventory (BDI). Further, they found that women who were initially the most distressed continued to score high on the GHQ and the BDI 1 year after experiencing the loss, in contrast to women who reported lower levels of distress, which continued to decline 3 months, 6 months, and 1 year after loss (Lok et al., 2010). Indeed, it is the recurrent nature of RPL can intensify this negative impact on mental health.

Emerging literature has therefore investigated the role of recurrent pregnancy loss on mental health outcomes such as stress, anxiety, and depression, as well as quality of life.

For example, a prospective case-control study conducted in Nigeria (Eleje et al., 2023) investigated differences in depression, stress, and anxiety symptoms among pregnant women with and without history of RPL. Using the Depression, Anxiety and Stress Scale, authors found that RPL was associated with moderate to severe levels of depression, anxiety and stress (p < .001). Further associations were found between high levels of stress and adverse outcomes such as preterm labour and repeat miscarriage (Eleje et al., 2023).

These findings are in line with Gao et al. (2020), who conducted a cross-sectional study in China to investigate the prevalence of anxiety and depression in a sample of 278 pregnant women with a history of RPL. Using self-report measures such as the Edinburgh Postnatal Depression Scale and the Self-rating Anxiety Scale, the authors found that 45% of their sample experienced anxiety, while 37% reported depressive symptomology. 10.13039/100014337Furthermore, they also found that low to moderate levels of social support, and a high school or below level of education were predictive of anxiety and depression symptoms(Gao et al., 2020).

Furthermore, research has demonstrated that depressive, anxiety and stress symptomology among pregnant women with RPL changes across the three trimesters of pregnancy. Qu et al. (2021) recruited 166 pregnant women with history of recurrent miscarriage, and completed mental health assessments at 6–12 weeks, 20–24 weeks and 32–36 weeks of gestation. They found that depression and anxiety prevalence was highest in early pregnancy and reduced during middle and late pregnancy. Finally, they found that social support was correlated with depression and anxiety throughout pregnancy. Data from UK Baby loss charity Tommy's shows that most miscarriages occur in the first 12 weeks, and this study has demonstrated that poor mental health is highest during the first trimester, when there is a fear of losing the unborn baby (Tommy's,n.d).

Tommy's has also shared results from a research study demonstrating that risk of recurrent pregnancy loss decreases as a woman's pregnancy progresses. More specifically, among a sample of 300 women with RPL, those who saw a heartbeat at their 6-week pregnancy scan had a 78% chance of their pregnancy continuing, which went up to 98% at their 8-week scan (Tommy’s,n.d). This further reiterates that mental health support should be particularly targeted at the first trimester in the circumstance of women with RPL history.

In addition to mood, anxiety and stress, another study has investigated the quality-of-life differences in pregnant women with, and without RPL (Abbaspoor et al., 2016). Specifically, the authors found that pregnant women with history of RPL had poorer outcomes across dimensions of quality of life such as ‘Body Pain’, ‘Social Functioning’, ‘Emotional Role Limitations’, and ‘Physical Functioning’ (Abbaspoor et al., 2016). These findings were in line with another study conducted by Tavoli et al., which compared a sample of women (N = 105) with RPL and healthy controls (N = 105) and found that women with recurrent miscarriage reported lower quality of life across all domains except for physical functioning.

Providing adequate mental health support is a key component that should be integrated into the treatment plan of managing RPL. This has been identified by Donegan and colleagues. Specifically, results of their integrative review highlight the need to provide counselling and care pathways specific to birthing people and their families in a subsequent pregnancy following loss. Further antenatal clinic appointments, pregnancy monitoring, and ongoing psychological support should be provided to families expecting after RPL, in order to mitigate the impact of stress and poor mental health through the prenatal period (Donegan et al., 2023)

### 3.1 Effect of RPL on non-birthing partners

Most research investigating the effect of RPL on mental health focuses on the birthing partner, most often women. Some studies have taken the non-birthing partner into account, investigating the impact of RPL on their mental health. Hedegaard et al. (2021) conducted a cross-sectional study to investigate the prevalence of depression and stress among both men (N = 281) and women (N = 412) with RPL. They found that 1.8% of men in their sample had moderate to severe levels of depression, comparable with 2% of men in the comparison group. Among the women, this prevalence was 8.3% in the RPL history group, compared with 2.2% in the comparison group. This study therefore highlighted that prevalence of depressive symptomatology did not differ in non-birthing partners who had history of RPL.

Due et al. (2017) synthesised the literature on men's experiences of RPL and found that men have less intense levels of negative psychological outcomes compared to women, however they are more likely to engage in behaviours such as increased alcohol consumption. Interestingly, their qualitative findings highlighted that men believed that their role is primarily to provide support to their birthing partner, and they potentially felt overlooked and marginalised in comparison.

## 4 Pregnancy loss and maternal foetal attachment

Given the negative psychological outcomes of pregnancy loss discussed in section 3, these might in turn affect attachment in subsequent pregnancies due to the fear of experiencing another loss. As attachment is an essential component that underpins overall offspring development, it is vital to understand how attachment might be affected in the context of RPL. While there is not a plethora of research looking at attachment in pregnant females with multiple pregnancy losses, this outcome has indeed been investigated in the context of loss.

For example, Armstrong and Hutti (1998) investigated differences in antenatal attachment among women who experienced late pregnancy loss and primiparous women of similar gestational age. Using the Prenatal Attachment Inventory, they found that the pregnancy loss group of women had significantly lower levels of attachment compared with the other group.

Tsartsara and Johnson (2006) assessed the impact of miscarriage history on pregnant women's maternal foetal attachment across the 1st and 3rd trimesters, and utilised the Maternal Antenatal Attachment Scale, wherein the minimum and maximum scores are 19 and 95. They found that the mean first trimester score was 58.4 (SD = 6.3). This was lower than the mean first trimester score (M = 71.5, SD = 10.3) among participants without miscarriage history. In the third trimester, the mean score in the miscarriage history group increased (M = 80.8 SD = 3.1) (Tsartsara and Johnson, 2006). As evidence shows that miscarriage risk decreases as the pregnancy progresses, results from this study show that women become more attached to their unborn baby as their pregnancy progresses, potentially as they are less worried of experiencing another loss.

## 5 Conclusions and directions for future research

While some aspects of this topic have been studied for decades now, there continues to remain a gap in the literature. Much of aetiological research is focused on mechanisms in the gestational parent. More studies delving into potential mechanisms arising from the non-gestational parent could share valuable insights into this complex disorder. Additionally, research into unexplained RPL is a vital component to better understand treatment pathways and provide personalized treatment recommendations on a case-by-case basis.

The next steps are to understand how it affects the non-birthing partner, in heterosexual relationships as well as LGBTQ + couples, and create avenues of support for non-birthing parents. RPL's recurrent nature can most often intensify poor mental health outcomes. This can have negative impacts on both maternal mental health and offspring outcomes in subsequent pregnancies, such as lower levels of attachment, increased pregnancy specific anxiety, and potential developmental outcomes. While specialist perinatal mental health services do provide support for pregnancy loss, additional assistance must be provided to couples experiencing RPL. Given that most losses occur in the first trimester, and research shows that mental health is at its lowest level in the first trimester in women with RPL, support should begin as soon as pregnancy is confirmed, and should continue until birth.

## Competing interests declaration

The author declares that there are no conflicts of interest.

## Funding

RL's work is supported by the HappyMums Consortium Project, funded by the 10.13039/100019637European Commission's Horizon Scheme.

## CRediT authorship contribution statement

**Riddhi A Laijawala:** Writing – original draft, Conceptualization.

## Declaration of competing interest

The authors declare that they have no known competing financial interests or personal relationships that could have appeared to influence the work reported in this paper.

## Data Availability

No data was used for the research described in the article.

## References

[bib1] Abbas A., Tripathi P., Naik S., Agrawal S. (2004). Analysis of human leukocyte antigen (HLA)-G polymorphism in normal women and in women with recurrent spontaneous abortions. Eur. J. Immunogenet..

[bib2] Abbaspoor Z., Razmju P.S., Hekmat K. (2016). Relation between quality of life and mental health in pregnant women with prior pregnancy loss. J. Obstet. Gynaecol. Res..

[bib3] Ali S., Majid S., Niamat Ali Md, Taing S. (2020). Evaluation of T cell cytokines and their role in recurrent miscarriage. Int. Immunopharm..

[bib4] American Society for Reproductive Medicine (2012). Evaluation and treatment of recurrent pregnancy loss: a committee opinion. Fertil. Steril..

[bib6] Armstrong D., Hutti M. (1998). Pregnancy after perinatal loss: the relationship between anxiety and prenatal attachment. J. Obstet. Gynecol. Neonatal Nurs..

[bib7] Bansal A.S. (2010). Joining the immunological dots in recurrent miscarriage. Am. J. Reprod. Immunol..

[bib8] Bender Atik R., Christiansen O.B., Elson J., Kolte A.M., Lewis S., Middeldorp S., Nelen W., Peramo B., Quenby S., Vermeulen N., Goddijn M., The ESHRE Guideline Group on RPL (2018). ESHRE guideline: recurrent pregnancy loss. Human Reproduction Open.

[bib10] Cavalcante M.B., Cavalcante C.T., de M.B., Sarno M., da Silva A.C.B., Barini R. (2020). Antinuclear antibodies and recurrent miscarriage: systematic review and meta-analysis. Am. J. Reprod. Immunol..

[bib11] Che D., Fang Z., Pi L., Xu Y., Fu L., Zhou H., Gu X. (2021). The SERPINA4 rs2070777 AA genotype is associated with an increased risk of recurrent miscarriage in a southern Chinese population. Int. J. Wom. Health.

[bib12] Chen S., Yang G., Wu P., Sun Y., Dai F., He Y., Qian H., Liu Y., Shi G. (2020). Antinuclear antibodies positivity is a risk factor of recurrent pregnancy loss: a meta-analysis. Semin. Arthritis Rheum..

[bib13] Comba C., Bastu E., Dural O., Yasa C., Keskin G., Ozsurmeli M., Buyru F., Serdaroglu H. (2015). Role of inflammatory mediators in patients with recurrent pregnancy loss. Fertil. Steril..

[bib14] Daher S., Denardi K. de A.G., Blotta M.H.S.L., Mamoni R.L., Reck A.P.M., Camano L., Mattar R. (2004). Cytokines in recurrent pregnancy loss. Proceedings of the HIPPOKRATION Congress on Reproductive Immunology.

[bib15] Dimitriadis E., Menkhorst E., Saito S., Kutteh W.H., Brosens J.J. (2020). Recurrent pregnancy loss. Nat. Rev. Dis. Prim..

[bib16] Donegan G., Noonan M., Bradshaw C. (2023). Parents experiences of pregnancy following perinatal loss: an integrative review. Midwifery.

[bib17] Dosiou C., Giudice L.C. (2005). Natural killer cells in pregnancy and recurrent pregnancy loss: endocrine and immunologic perspectives. Endocr. Rev..

[bib18] Due C., Chiarolli S., Riggs D.W. (2017). The impact of pregnancy loss on men's health and wellbeing: a systematic review. BMC Pregnancy Childbirth.

[bib19] El Hachem H., Crepaux V., May-Panloup P., Descamps P., Legendre G., Bouet P.-E. (2017). Recurrent pregnancy loss: current perspectives. Int. J. Wom. Health.

[bib20] Eleje G.U., Oguejiofor C.B., Oriji S.O., Ekwuazi K.E., Ugwu E.O., Igbodike E.P., Malachy D.E., Nwankwo E.U., Onah C.E., Ugboaja J.O., Ikechebelu J.I., Nwagha U.I. (2023). Depression, anxiety, and stress and adverse pregnancy outcomes in pregnant women with history of recurrent pregnancy loss in Nigeria. Int. J. Psychiatr. Med..

[bib21] Ford H.B., Schust D.J. (2009). Recurrent pregnancy loss: etiology, diagnosis, and therapy. Rev Obstet Gynecol.

[bib22] Gao L., Qu J., Wang A.Y. (2020). Anxiety, depression and social support in pregnant women with a history of recurrent miscarriage: a cross-sectional study. J. Reprod. Infant Psychol..

[bib23] Ghaebi M., Nouri M., Ghasemzadeh A., Farzadi L., Jadidi-Niaragh F., Ahmadi M., Yousefi M. (2017). Immune regulatory network in successful pregnancy and reproductive failures. Biomed. Pharmacother..

[bib24] Giannubilo S.R., Landi B., Pozzi V., Sartini D., Cecati M., Stortoni P., Corradetti A., Saccucci F., Tranquilli A.L., Emanuelli M. (2012). The involvement of inflammatory cytokines in the pathogenesis of recurrent miscarriage. Cytokine.

[bib25] Goldberg D.P., Hillier V.F. (1979). A scaled version of the general health Questionnaire. Psychol. Med..

[bib26] Grigoriadis S., Graves L., Peer M., Mamisashvili L., Tomlinson G., Vigod S.N., Dennis C.-L., Steiner M., Brown C., Cheung A., Dawson H., Rector N.A., Guenette M., Richter M. (2018). Maternal anxiety during pregnancy and the association with adverse perinatal outcomes: systematic review and meta-analysis. J. Clin. Psychiatry.

[bib27] Grimstad F., Krieg S. (2016). Immunogenetic contributions to recurrent pregnancy loss. J. Assist. Reprod. Genet..

[bib28] Hedegaard S., Landersoe S.K., Olsen L.R., Krog M.C., Kolte A.M., Nielsen H.S. (2021). Stress and depression among women and men who have experienced recurrent pregnancy loss: focusing on both sexes. Reprod. Biomed. Online.

[bib29] Hviid T.V.F. (2006). HLA-G in human reproduction: aspects of genetics, function and pregnancy complications. Hum. Reprod. Update.

[bib30] Katano K., Suzuki S., Ozaki Y., Suzumori N., Kitaori T., Sugiura-Ogasawara M. (2013). Peripheral natural killer cell activity as a predictor of recurrent pregnancy loss: a large cohort study. Fertil. Steril..

[bib31] Klier C.M., Geller P.A., Ritsher J.B. (2002). Affective disorders in the aftermath of miscarriage: a comprehensive review. Arch. Wom. Ment. Health.

[bib32] Kwak-Kim J., Yang K.M., Gilman-Sachs A. (2009). Recurrent pregnancy loss: a disease of inflammation and coagulation. J. Obstet. Gynaecol. Res..

[bib33] Laird S.M., Tuckerman E.M., Cork B.A., Linjawi S., Blakemore A.I.F., Li T.C. (2003). A review of immune cells and molecules in women with recurrent miscarriage. Hum. Reprod. Update.

[bib34] Li Q., Chen S., Dong X., Fu S., Zhang T., Zheng W., Tian Y., Huang D. (2023). The progress of research on genetic factors of recurrent pregnancy loss. Genetics Res..

[bib35] Lok I.H., Yip A.S.-K., Lee D.T.-S., Sahota D., Chung T.K.-H. (2010). A 1-year longitudinal study of psychological morbidity after miscarriage. Fertil. Steril..

[bib37] Marozio L., Nuzzo A.M., Gullo E., Moretti L., Canuto E.M., Tancredi A., Goia M., Cosma S., Revelli A., Rolfo A., Benedetto C. (2023). Immune checkpoints in recurrent pregnancy loss: new insights into a detrimental and elusive disorder. Int. J. Mol. Sci..

[bib38] Morelli S.S., Mandal M., Goldsmith L.T., Kashani B.N., Ponzio N.M. (2015). The maternal immune system during pregnancy and its influence on fetal development. Res. Rep. Biol..

[bib39] Muncey W., Scott M., Lathi R.B., Eisenberg M.L. (2024). The paternal role in pregnancy loss. Andrology.

[bib40] NHS UK (2022). Antiphospholipid syndrome (APS). NHS. Int. J. Nurs. Pract..

[bib41] Qu J., Weng X., Gao L. (2021).

[bib42] Rai R., Regan L. (2006). Recurrent miscarriage. Lancet.

[bib44] Regan L., Rai R., Saravelos S., Li T.-C., the Royal College of Obstetricians and Gynaecologists (2023). Recurrent MiscarriageGreen-top guideline No. 17. BJOG An Int. J. Obstet. Gynaecol..

[bib45] Saccone G., Berghella V., Maruotti G.M., Ghi T., Rizzo G., Simonazzi G., Rizzo N., Facchinetti F., Dall'Asta A., Visentin S., Sarno L., Xodo S., Bernabini D., Monari F., Roman A., Eke A.C., Hoxha A., Ruffatti A., Schuit E., Martinelli P. (2017). Antiphospholipid antibody profile based obstetric outcomes of primary antiphospholipid syndrome: the PREGNANTS study. Am. J. Obstet. Gynecol..

[bib46] Santos T. da S., Ieque A.L., de Carvalho H.C., Sell A.M., Lonardoni M.V.C., Demarchi I.G., de Lima Neto Q.A., Teixeira J.J.V. (2017). Antiphospholipid syndrome and recurrent miscarriage: a systematic review and meta-analysis. J. Reprod. Immunol..

[bib47] Sawyer K.M. (2021). The role of inflammation in the pathogenesis of perinatal depression and offspring outcomes. Brain, Behavior Immunity Health.

[bib48] Stout M.J., Cao B., Landeau M., French J., Macones G.A., Mysorekar I.U. (2015). Increased human leukocyte antigen-G expression at the maternal–fetal interface is associated with preterm birth. J. Matern. Fetal Neonatal Med..

[bib49] Sultana Shehnaz, Nallari Pratibha, Ananthapur Venkateshwari (2020). Recurrent pregnancy loss (RPL): an overview. Recurrent pregnancy loss (RPL): an overview. J. Women’s Health Dev..

[bib50] Tavoli Z., Mohammadi M., Tavoli A., Moini A., Effatpanah M., Khedmat L., Montazeri A. (2018). Quality of life and psychological distress in women with recurrent miscarriage: a comparative study. Health Qual. Life Outcome.

[bib51] Thomsen C.K., Steffensen R., Nielsen H.S., Kolte A.M., Krog M.C., Egerup P., Larsen E.C., Hviid T.V., Christiansen O.B. (2021). HLA-DRB1 polymorphism in recurrent pregnancy loss: new evidence for an association to HLA-DRB1∗07. J. Reprod. Immunol..

[bib52] Tommy’s. Miscarriage statistics and rates in the UK. (n.d.). https://www.tommys.org/baby-loss-support/miscarriage-information-and-support/miscarriage-statistics#:∼:text=Oneresearchstudyofmore,chanceofthepregnancycontinuing.

[bib53] Tsartsara E., Johnson M.P. (2006). The impact of miscarriage on women's pregnancy-specific anxiety and feelings of prenatal maternal–fetal attachment during the course of a subsequent pregnancy: an exploratory follow-up study. J. Psychosom. Obstet. Gynecol..

[bib54] van Dijk M.M., Kolte A.M., Limpens J., Kirk E., Quenby S., van Wely M., Goddijn M. (2020). Recurrent pregnancy loss: diagnostic workup after two or three pregnancy losses? A systematic review of the literature and meta-analysis. Hum. Reprod. Update.

[bib55] Vinatier D., Dufour P., Cosson M., Houpeau J.L. (2001). Antiphospholipid syndrome and recurrent miscarriages. Eur. J. Obstet. Gynecol. Reprod. Biol..

[bib56] Walter I.J., Klein Haneveld M.J., Lely A.T., Bloemenkamp K.W.M., Limper M., Kooiman J. (2021). Pregnancy outcome predictors in antiphospholipid syndrome: a systematic review and meta-analysis. Autoimmun. Rev..

